# Mechanical Strains Induced in Osteoblasts by Use of Point Femtosecond Laser Targeting

**DOI:** 10.1155/IJBI/2006/21304

**Published:** 2006-12-06

**Authors:** Charles Cranfield, Ze'ev Bomzon, Daniel Day, Min Gu, Sarah Cartmell

**Affiliations:** ^1^ Centre for Micro-Photonics, Faculty of Engineering and Industrial Sciences, Swinburne University of Technology, Hawthorn, VIC 3122, Australia; ^2^ Faculty of Medicine, Technion-Israel Institute of Technology, Haifa 32000, Israel,; ^3^ Institute for Science and Technology in Medicine, Keele University, Keele, Staffordshire ST4 7QB, UK

## Abstract

A study demonstrating how ultrafast laser radiation stimulates osteoblasts is presented. The study employed a custom made optical system that allowed for simultaneous confocal cell imaging and targeted femtosecond pulse laser irradiation. When femtosecond laser light was focused onto a single cell, a rise in intracellular Ca^2+^ levels was observed followed by contraction of the targeted cell. This contraction caused deformation of neighbouring cells leading to a heterogeneous strain field throughout the monolayer. Quantification of the strain fields in the monolayer using digital image correlation revealed local strains much higher than threshold values typically reported to stimulate extracellular bone matrix production in vitro. This use of point targeting with femtosecond pulse lasers could provide a new method for stimulating cell activity in orthopaedic tissue engineering.


Hindawi Publishing Corporation
International Journal of Biomedical Imaging
Volume 2006, Article ID 21304, Pages 1–6
DOI 10.1155/IJBI/2006/21304

### Mechanical Strains Induced in Osteoblasts by Use of Point Femtosecond Laser Targeting

**Charles Cranfield,[1] Ze'ev Bomzon,[1,2] Daniel Day,[1] Min Gu,[1] and Sarah Cartmell[3]**

[1] *Centre for Micro-Photonics, Faculty of Engineering and Industrial Sciences, Swinburne University of Technology, Hawthorn, VIC 3122, Australia*
[2] *Faculty of Medicine, Technion-Israel Institute of Technology, Haifa 32000, Israel*
[3] *Institute for Science and Technology in Medicine, Keele University, Keele, Staffordshire ST4 7QB, UK*

Received 11 May 2006; Revised 3 September 2006; Accepted 17 September 2006

Recommended for Publication by Lizhi Sun

A study demonstrating how ultrafast laser radiation stimulates osteoblasts is presented. The study employed a custom made optical system that allowed for simultaneous confocal cell imaging and targeted femtosecond pulse laser irradiation. When femtosecond laser light was focused onto a single cell, a rise in intracellular $Ca^{2+}$ levels was observed followed by contraction of the targeted cell. This contraction caused deformation of neighbouring cells leading to a heterogeneous strain field throughout the monolayer. Quantification of the strain fields in the monolayer using digital image correlation revealed local strains much higher than threshold values typically reported to stimulate extracellular bone matrix production in vitro. This use of point targeting with femtosecond pulse lasers could provide a new method for stimulating cell activity in orthopaedic tissue engineering.

Copyright © 2006 Charles Cranfield et al. This is an open access article distributed under the Creative Commons Attribution License, which permits unrestricted use, distribution, and reproduction in any medium, provided the original work is properly cited.

### 1. INTRODUCTION

In recent years the effect of laser radiation on cells has become the topic of much research. It has been shown that laser radiation can be used for cellular microsurgery [1], disruption and inactivation of cellular organelles [2], to induce photodamage in cells [3], and to induce changes in intracellular calcium ($Ca^{2+}$) levels [3–5]. It has also been demonstrated that a focused period of femtosecond pulses can be used for tissue dissection [6], cell microinjection [7, 8], and cell transfection [9].

One of the areas in which stimulation of cells with femtosecond laser radiation might find extended applications is orthopaedic tissue engineering. Tissue engineering aims to create biologically functional tissue substitutes. Generally this is done by seeding cells on a biocompatible scaffold and cultivating within a bioreactor. The bioreactor provides the appropriate environment and stimuli to the cellular construct so that tissue integrity is optimised prior to implantation. It is widely recognised that successful tissue engineering requires not only coordinate biochemical stimulus of the cell, but also physical stimulus of the cell. In particular, engineering of load-bearing tissues such as bones requires mechanical stimulation of the constructs in order to optimise the engineered scaffold's mechanical properties [10].

A variety of methods have been used to mechanically stimulate bone cells in artificial tissue scaffolds. These methods include compression rigs [11] and shear flow chambers [12]. Although these methods are extremely useful, one of their limitations is that they apply a gross mechanical strain to the entire structure. Thus it is difficult to control the stimulation of single cells within specific regions of the construct. This kind of stimulation is desirable as it allows for control of the engineered tissue's properties on a highly localised scale. Here we present evidence that focused femtosecond (fs) laser radiation can be used to stimulate single osteoblastic cells in monolayer. Since laser radiation can be controlled with high spatial and temporal resolution, the technology described here would prove useful for bone tissue engineering. This technique has the potential to be incorporated into a sophisticated bioreactor for the culturing of bone tissue.

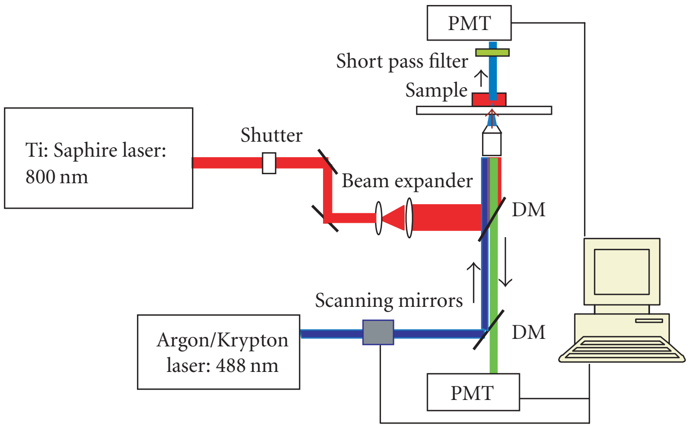

Figure 1: Diagrammatic representation of the Olympus confocal microscope adapted for simultaneous imaging and fs pulse laser exposure. This system allows for simultaneous imaging of cells being targeted with the femtosecond pulsed laser. DM = dichroic mirror. PMT = photomultiplier tube.

### 2. METHODS

### 2.1. Cell culture

MC3T3-E1 (3T3) murine osteoblast-like cells were seeded onto autoclaved round 40 mm diameter coverslips at a density of 100 000 cells per coverslip. The 3T3 cells were grown in Dubecco's modified eagle medium (DMEM) supplemented with HEPES (20 mM), 10% fetal bovine serum, L-glutamine (200 mM), and penicillin-streptomycin (1%) (SIGMA-ALDRICH, Castle Hill, NSW, Australia) for 24 hours prior to imaging.

Prior to laser stimulation and imaging, cells were incubated with 1-2 $\mu$M fluo-3 AM (SAPPHIRE BIOSCIENCE, Redfern, NSW, Australia) for 1 hour at room temperature in DMEM and 20 mM HEPES without supplements for $Ca^{2+}$ monitoring. Cells were kept out of the light for the duration of fluo-3 incubation. After fluo-3 dye incubation, the cells were washed and immersed in fresh DMEM (with no supplements) ready for imaging.

### 2.2. Imaging

Imaging at 37°C was performed with the assistance of a Focht Chamber System (FCS2) (BIOPTECHS, Butler, Pa, USA). This allowed cells to be imaged on the inverted microscope, whilst simultaneously being perfused with media that is kept at a constant 37°C (±0.1°C). Perfusion media consisted of DMEM with HEPES (20 mM), but without any other supplements. The media was continually bubbled with carbogen gas to maintain a stable pH. Cells in regions of high cell confluence were chosen as targets for laser irradiation.

Delivery of the femtosecond pulse laser to cells was carried out using an adapted Olympus FV300 confocal microscope (OLYMPUS AUSTRALIA, Mount Waverly, VIC, Australia). The shutter-controlled femtosecond laser line was expanded to exceed the diameter of the back aperture of an Olympus 60 × 1.25 NA objective, and fed into the back port of an Olympus IX70 inverted microscope, where a short pass dichroic mirror was installed. This dichroic allowed for simultaneous fluorescence excitation using the scanned 488 nm line of the krypton: argon ion laser of the FV300 confocal microscope whilst simultaneously allowing for the femtosecond pulse laser to target the sample at a fixed point.

The source for the femtosecond beam was a Spectra Physics MaiTai Titanium: sapphire femtosecond pulsed laser which produces 80 fs pulses at a repetition rate of 80 MHz and an average power of 950 mW (SPECTRA PHYSICS, Mountain View, Calif, USA). The MaiTai has a tuneable wavelength range from 730 nm to 870 nm, but for these experiments the wavelength was set at 800 nm. The femtosecond beam was passed through a Uniblitz LS6 mechanical shutter (UNIBLITZ, USA). Shutter time was set to 500 ms for the experiment. The beam passed through a neutral density filter wheel before it was expanded and directed by use of lenses and mirrors through the rear of an Olympus IX71 microscope, where it is was then directed through an Olympus 60 × 1.25 oil objective into the sample. The power at the back aperture of the objective was measured to be between 8 mW and 15 mW. In order to visualise the transmitted image whilst laser exposure was occurring, a short pass filter was placed in the transmission path of the confocal microscope. Figure 1 shows a diagram of the system used for simultaneous imaging and femtosecond irradiation.

### 2.3. Strain and displacement mapping

Cellular strains and displacement were computed using digital image correlation (DIC), which is a pattern matching technique that allows measurement of displacements with subpixel resolution from sequences of images. This study used an algorithm previously described [13–15]. The algorithm was realized using MATLAB 7.0 (The MathWorks,

Natick, Ma, USA), and was applied to the sequences of fluorescent images associated with the fluo-3 fluorophore. Prior to applying DIC, a Wiener adaptive noise reduction filter with a kernel size of 10 × 10 pixels was applied to all images in a sequence. Next, approximately 700 *regions of interests* (ROIs) of size 49 × 49 pixels were randomly placed throughout the image. DIC was then applied to measure the displacement of the centre of each ROI throughout the sequence of images. This was accomplished by comparing the first image to all other images in the sequence. In order to calculate the strain components from the displacement data, a thin plate smooth spline was fitted to the measured displacements. Then Delaunay triangulation was applied to draw the smallest possible set of triangles to connect the centres of all ROIs. The displacements of the vertices of these triangles as measured from the thin plate spline defined a set of three linear equations for each triangle. These equations were solved to yield the average deformation tensor within each triangle,

$$\mathbf{F} = \begin{bmatrix} \dfrac{dx}{dX} & \dfrac{dx}{dY} \\ \dfrac{dy}{dX} & \dfrac{dy}{dY} \end{bmatrix}. \quad (1)$$

In (1) $(X, Y)$ denote coordinates in the reference image (first image in the sequence), and $(x, y)$ denote coordinates in the deformed image. The average Lagrangian strain, $\mathbf{E}$, within each triangle was then calculated as

$$\mathbf{E} = 0.5(\mathbf{F}^T\mathbf{F} - \mathbf{I}), \quad (2)$$

where $\mathbf{F}^T$ is the transpose of $\mathbf{F}$, and $\mathbf{I}$ is the unity tensor. Next, the eigenvalues of the strain tensor were found to yield the principal strains (E1, E2) within each triangle.

The principal strains were interpolated to yield an estimate of the strain fields throughout the field of view. The strain fields were smoothed with a moving average filter with a kernel size of 20 × 20 pixels to filter noise. Visualization of the distribution of strains throughout the field of view was obtained by creating colour-maps depicting the spatial distribution of strains. These colour-maps were created so that blue indicates compressive (negative) strains and red indicates tensile (positive) strains.

### 3. RESULTS

In order to demonstrate that targeting cells with the femtosecond pulsed laser was having a visible effect it was necessary to load cells with a marker that could rapidly detect cellular changes. One of the most effective methods in accomplishing this was to use calcium ion fluorophores that can rapidly detect any alterations in local calcium levels caused by the laser. Targeting of individual cells with fs pulse laser irradiation caused an instant transient rise in intracellular $Ca^{2+}$ levels. In almost all cases it was possible to target cells with the fs pulse laser multiple times to get a repeated increase in intracellular $Ca^{2+}$ (Figure 2). $Ca^{2+}$ would normally return to baseline levels approximately 2 min after point laser irradiation.

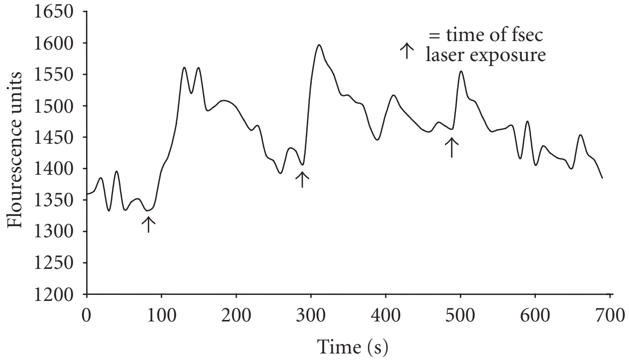

Figure 2: Graphical representation of the change in $Ca^{2+}$ levels as measured within a region of interest surrounding a typical target cell. Repeated targeting of the cell induces repeated calcium spikes. Arrows indicate the time-points of the exposure to 500 ms of fs pulse laser radiation.

As the calcium load returned to resting levels a cellular contraction was observed in nearly all cases ($n = 11$). The displacements of the individual target cell then induced a radial displacement pattern from the surrounding cells in the monolayer (Figure 3). These displacements were measured using DIC. Calculation of the strain field from the measured displacements was performed using the procedure described in [13, 14]. The calculations revealed a heterogeneous strain field within the monolayer. The principle strains around the targeted cell were of the order of 20% (Figure 4). The magnitude of the strains decreased with distance from the targeted cell. However, even at a distance of about 5 cell lengths ($\sim 50\,\mu$m) significant strains with a magnitude of more than 5% were measured. This level of deformation is larger than strain levels reported to stimulate bone mineralisation in vitro [16].

### 4. DISCUSSION

This study shows that focused ultrafast laser radiation can stimulate murine MC3T3-E1 (3T3) osteoblast-like cells. Femtosecond irradiation of cells loaded with fluo-3 AM causes a transient rise in intracellular $Ca^{2+}$ levels, which is accompanied by contraction of the irradiated cell. This contraction causes a heterogeneous strain field in the surrounding monolayer. The resulting strains are of the order of 20% near the target cell, and gradually decrease with distance from the cell. Cell strains of as little as 0.8%–1% have been reported to upregulate bone mineralisation related transcriptional activity [17]. Osteoblasts have also been shown to upregulate osteopontin production 2.8 fold after a tensile strain application of up to just 10% [18]. Furthermore, it has been shown that an increase in intracellular $Ca^{2+}$ in osteoblasts also has effects on bone cell stimulation [19]. Thus femtosecond lasers might be useful for stimulating bone mineralisation in tissue constructs by artificially inducing increases in intracellular $Ca^{2+}$, as well as causing cellular deformation. In the past continuous wave lasers have been used to enhance

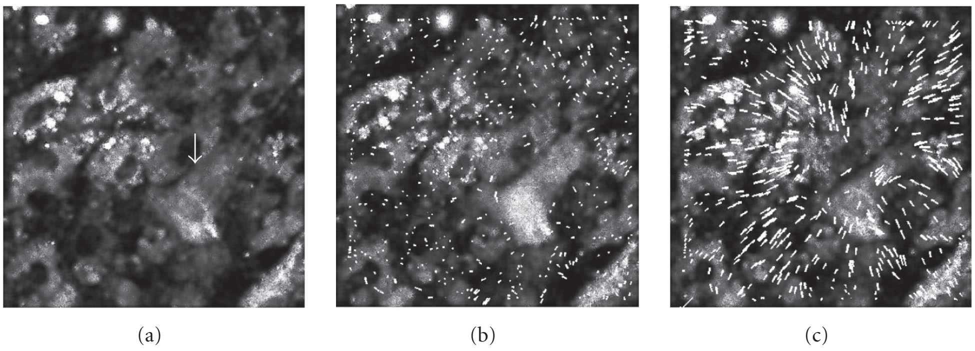

Figure 3: Images of 3T3 cells loaded with fluo-3 AM: (a) before fs pulse laser targeting ($t = -150$ s); (b) 20 s after fs pulse laser targeting ($t = 20$); and 2 min after that ($t = 140$). Images (b) and (c) are overlaid with arrows which represent cell displacements brought about by the target cell contracting. Arrows are 3 times larger than actual displacements. A clear rise in intracellular fluorescence due to increased $Ca^{2+}$ can be seen in the targeted cell at $t = 20$ (b), which has subsided by $t = 140$ (c). Image scale: 160 $\mu$m × 160 $\mu$m.

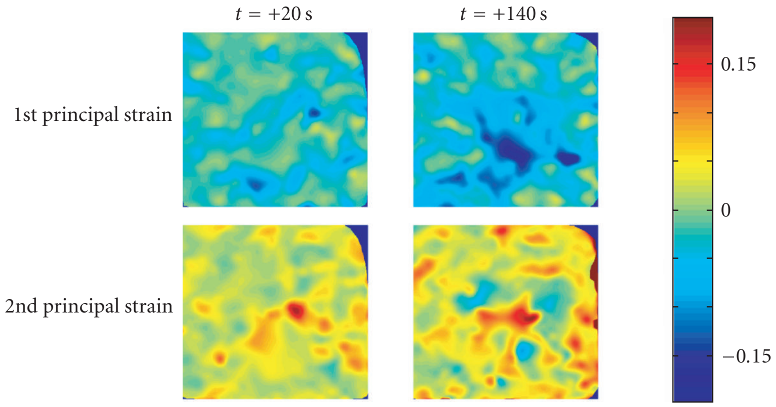

Figure 4: A comparison of the first and second principal strains comparing $t = 20$ and $t = 140$ using the same data as that of Figure 3. The red colours indicate positive strains (tension), whilst the blue colours indicate negative strains (compression). Image scale: 160 $\mu$m × 160 $\mu$m.

bone repair and bone stimulation [19, 20]. However using femtosecond pulse lasers in the near infrared for manipulating cells might be preferable because of the increased penetration depth, highly localized nonlinear photo-damage, and limited heat transfer to samples.

This project utilised a novel system that allowed for simultaneous confocal imaging and femtosecond stimulation of the cells with parallel beams. This is an improvement on a previously described system, in which confocal imaging and femtosecond stimulation were achieved using counterpropagating beams [5]. The advantage of this system over the previous systems is that it allows for confocal imaging and femtosecond stimulation whilst cells remain in an enclosed and sterile environment such as a perfusion chamber or bioreactor. It is more difficult to obtain the same level of sterility with counterpropagating beams, as the system in this configuration requires access points for two objectives.

Cellular deformation in response to fs pulse laser irradiation was quantified using digital image correlation (DIC), a computational technique for analysing movement and distortion within pairs of images. DIC has previously been used for measuring deformation in articular cartilage [21, 22] trabecular bone, [23], compressed chondrocytes [14], and intracellular strains in mechanically stimulated smooth muscle cells [16]. Thus DIC has become an established technique for biomechanical measurements at the tissue and cellular levels. The ability to measure displacements and strains on a highly localised level can provide a quantitative measure for evaluating local mechanical properties in engineered tissue constructs. Thus this information could be used to locate regions within the construct in which cellular stimulation is required to modify the local construct properties.

In this study we monitored alterations in $Ca^{2+}$ as a result of laser stimulation. However calcium ions are not the only intracellular messenger that can be monitored for mechanotransduction processes using this system; upregulation of nitric oxide can be monitored using commercial fluorophores such as DAF-FM, and alterations in structural proteins as a result of laser-induced mechanotransduction can be assessed using appropriate green fluorescent protein type targeted vectors. Using the femtosecond pulse laser to target the extracellular matrix, individual microtubules, or even microfilaments inside cells, thereby inducing localised displacements, would help in the understanding of the tensegrity [24] of cellular structure, and how this might be affecting the mechanotransduction signals of cells.

In summary, a novel system for stimulating osteoblasts in vitro has been presented. The system combines femtosecond pulse laser point targeting with confocal microscopy. This system has many potential applications in orthopaedic tissue engineering related research.

### ACKNOWLEDGMENTS

We like to thank Dr. Natalie Sims from St. Vincent's Institute, Melbourne, for the donation of the MC3T3-E1 cells. This project was financed by the Fluorescence Applications in Biotechnology and Life-sciences (FABLS) Network, which is an Australia Research Council/National Health & Medical Research Council funded network.

### REFERENCES

[1] W. Watanabe, N. Arakawa, S. Matsunaga, et al., "Femtosecond laser disruption of subcellular organelles in a living cell," *Optics Express*, vol. 12, no. 18, pp. 4203–4213, 2004.

[2] V. Venugopalan, A. Guerra III, K. Nahen, and A. Vogel, "Role of laser-induced plasma formation in pulsed cellular microsurgery and micromanipulation," *Physical Review Letters*, vol. 88, no. 7, Article ID 078103, 4 pages, 2002.

[3] H. J. Koester, D. Baur, R. Uhl, and S. W. Hell, "$Ca^{2+}$ fluorescence imaging with pico- and femtosecond two-photon excitation: signal and photodamage," *Biophysical Journal*, vol. 77, no. 4, pp. 2226–2236, 1999.

[4] N. I. Smith, K. Fujita, T. Kaneko, et al., "Generation of calcium waves in living cells by pulsed-laser-induced photodisruption," *Applied Physics Letters*, vol. 79, no. 8, pp. 1208–1210, 2001.

[5] D. Day, C. Cranfield, and M. Gu, "High-speed fluorescence imaging and intensity profiling of femtosecond-induced calcium transients," *International Journal of Biomedical Imaging*, vol. 2006, Article ID 93438, 6 pages, 2006.

[6] S. Sikder and R. W. Snyder, "Femtosecond laser preparation of donor tissue from the endothelial side," *Cornea*, vol. 25, no. 4, pp. 416–422, 2006.

[7] S. K. Mohanty, M. Sharma, and P. K. Gupta, "Laser-assisted microinjection into targeted animal cells," *Biotechnology Letters*, vol. 25, no. 11, pp. 895–899, 2003.

[8] W. Tao, J. Wilkinson, E. J. Stanbridge, and M. W. Berns, "Direct gene transfer into human cultured cells facilitated by laser micropuncture of the cell membrane," *Proceedings of the National Academy of Sciences of the United States of America*, vol. 84, no. 12, pp. 4180–4184, 1987.

[9] U. K. Tirlapur and K. König, "Cell biology: targeted transfection by femtosecond laser," *Nature*, vol. 418, no. 6895, pp. 290–291, 2002.

[10] A. J. El Haj, M. A. Wood, P. Thomas, and Y. Yang, "Controlling cell biomechanics in orthopaedic tissue engineering and repair," *Pathologie Biologie*, vol. 53, no. 10, pp. 581–589, 2005.

[11] M. A. Wood, Y. Yang, B. M. Thomas, and A. J. El Haj, "Using dihydropyridine-release strategies to enhance load effects in engineered human bone constructs," *Tissue Engineering*, vol. 12, no. 9, pp. 2489–2497, 2006.

[12] S. Cartmell, B. D. Porter, A. J. García, and R. E. Guldberg, "Effects of medium perfusion rate on cell-seeded three-dimensional bone constructs in vitro," *Tissue Engineering*, vol. 9, no. 6, pp. 1197–1203, 2003.

[13] Y. Wang and A. M. Cuitino, "Full-field measurements of heterogeneous deformation patterns on polymeric foams using digital image correlation," *International Journal of Solids and Structures*, vol. 39, no. 13-14, pp. 3777–3796, 2002.

[14] M. M. Knight, Z. Bomzon, E. Kimmel, A. M. Sharma, D. A. Lee, and D. L. Bader, "Chondrocyte deformation induces mitochondrial distortion and heterogeneous intracellular strain fields," *Biomechanics and Modeling in Mechanobiology*, vol. 5, no. 2-3, pp. 180–191, 2006.

[15] Z. Bomzon, M. M. Knight, D. L. Bader, and E. Kimmel, "Mitochondrial dynamics in chondrocytes and their connection to the mechanical properties of the cytoplasm," *Journal of Biomechanical Engineering*, vol. 128, no. 5, pp. 674–679, 2006.

[16] S. Hu, J. Chen, B. Fabry, et al., "Intracellular stress tomography reveals stress focusing and structural anisotropy in cytoskeleton of living cells," *American Journal of Physiology - Cell Physiology*, vol. 285, no. 5, pp. C1082–C1090, 2003.

[17] X. Chen, C. M. Macica, K. W. Ng, and A. E. Broadus, "Stretch-induced PTH-related protein gene expression in osteoblasts," *Journal of Bone and Mineral Research*, vol. 20, no. 8, pp. 1454–1461, 2005.

[18] K. D. Fong, S. M. Warren, E. G. Loboa, et al., "Mechanical strain affects dura mater biological processes: implications for immature calvarial healing," *Plastic and Reconstructive Surgery*, vol. 112, no. 5, pp. 1312–1327, 2003.

[19] T. Yaakobi, L. Maltz, and U. Oron, "Promotion of bone repair in the cortical bone of the tibia in rats by low energy laser (He-Ne) irradiation," *Calcified Tissue International*, vol. 59, no. 4, pp. 297–300, 1996.

[20] A. Stein, D. Benayahu, L. Maltz, and U. Oron, "Low-level laser irradiation promotes proliferation and differentiation of human osteoblasts in vitro," *Photomedicine and Laser Surgery*, vol. 23, no. 2, pp. 161–166, 2005.

[21] N. O. Chahine, C. C.-B. Wang, C. T. Hung, and G. A. Ateshian, "Anisotropic strain-dependent material properties of bovine articular cartilage in the transitional range from tension to compression," *Journal of Biomechanics*, vol. 37, no. 8, pp. 1251–1261, 2004.

[22] C. C.-B. Wang, N. O. Chahine, C. T. Hung, and G. A. Ateshian, "Optical determination of anisotropic material properties of bovine articular cartilage in compression," *Journal of Biomechanics*, vol. 36, no. 3, pp. 339–353, 2003.

[23] T. O. McKinley and B. K. Bay, "Trabecular bone strain changes associated with subchondral stiffening of the proximal tibia," *Journal of Biomechanics*, vol. 36, no. 2, pp. 155–163, 2003.

[24] D. E. Ingber, "Tensegrity: the architectural basis of cellular mechanotransduction," *Annual Review of Physiology*, vol. 59, pp. 575–599, 1997.

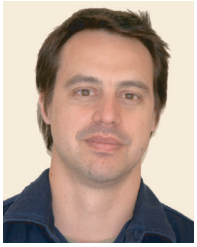

**Charles Cranfield** was born in Melbourne, Australia, in 1971. He received the B.S. degree with honours in pharmacology from Monash University, Melbourne, Australia, in 1996. This was followed by Ph.D. degree in biophysics from Swinburne University of Technology, Melbourne, Australia, in 2002. His research was into possible mobile telephone RF effects on calcium ion levels in human T lymphocytes. He was invited to discuss these findings to researchers at the Babraham Institute, Cambridge, UK. He then completed 2 years as a Postdoctoral Researcher in Professor Jon Dobson's Nanomagnetics Laboratory, Keele University, UK. His work there culminated in the discovery of biogenic magnetite in the nematode *C.* elegans. This research was the basis for an article in "Chemistry World" magazine in 2004 entitled "*As the magnetic worm turns; Magnetite reignites mobile phone radiation concerns.*" In 2004 he returned to Australia to take up a brief position at Prince Henry's Institute for Medical Research, Melbourne, investigating calcium responses in pituitary cells, before taking a position as Research Fellow of Biophotonics at the Centre for Micro-Photoincs, Swinburne University of Technology. More recently he accepted a Marie Curie transfer of Knowledge Fellowship at Tyndall Institute in Ireland.

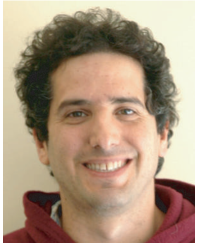

**Ze′ev Bomzon** received his Bachelors degree in mathematics and physics from the Technion-Israel Institute of Technology in 1999. In 2002 he completed his Masters degree at the same institute, and in 2005 he completed his Ph.D. degree at the Technion. In 2004 he was awarded the European Society of Biomechanics Student Award for his work. On completion of his studies he joined the Centre for Micro-Photonics at Swinburne University of Technology, Melbourne, Australia as a Visiting Postdoctoral Fellow. In 2006 he was awarded a Linkage International Fellowship from the Australian Research Council, as well as a Marie Curie Outgoing International Fellowship from the sixth European Framework. His research has focused on both optics and biomechanics. He has published sixteen papers in peer-reviewed journals. His current research focuses on the development of optical tools for studying cell mechanics.

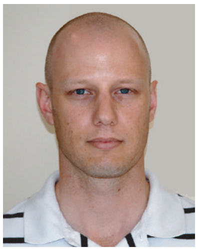

**Daniel Day** received his B.S. degree from Griffith University, Brisbane, Australia in 1996 with majors in physics and mathematics, and in 1997 received First Class Honours from Victoria University of Technology, Melbourne, Australia, in optics. He gained his Ph.D. degree from Swinburne University of Technology, Melbourne, Australia, in 2001, researching three-dimensional optical data storage. His Ph.D. thesis was "Three-dimensional bit optical data storage in a photorefractive polymer". Recently Daniel completed a Graduate Certificate in Entrepreneurship and Innovation at Swinburne University of Technology. In 1998 he received the Australian Optical Society Postgraduate Student Award for his research into three-dimensional optical data storage. He is a Member of the Australian Institute of Physics, Australian Optical Society, and the Optical Society of America. Since completing his Ph.D. degree, he has established the company 3DCD Technology Pty. Ltd. with Swinburne University which now holds several international patents in three-dimensional optical data storage. Currently he is a Research Fellow in the Centre for Micro-Photonics at Swinburne University of Technology, Melbourne, Australia, conducting research into three-dimensional microfluidics and fabrication of three-dimensional micro-environments for biological applications.

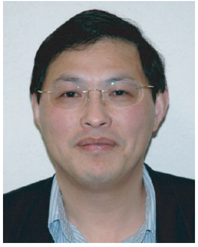

**Min Gu** gained a Ph.D. degree in optics from the Chinese Academy of Sciences in 1988. He was invited for the appointment of Professor (Chair) of Optoelectronics and Director of the Centre for Micro-Photonics at Swinburne University of Technology from 2000. In 2003 he became a Node Director of the Australian Research Council Centre of Excellence for Ultrahigh-bandwidth Devices for Optical Systems. He is a sole author of two standard reference books, "Principles of Three-Dimensional Imaging in Confocal Microscopes" and "Advanced Optical Imaging Theory". He published over 300 papers in photonic crystals, nanophotonics, micro/nanofabrication, confocal microscopy, laser tweezers, optoelectronic imaging through tissue-like turbid media, laser trapping microscopy, and three-dimensional optical data storage. He is a topical Editor of 2 international optics journals, a Member of the editorial board of another 4 international journals, and was the Guest Editor of Applied Optics and the International Journal of Optical Memory and Neural Networks. He is President and Regional Council Member of the International Society of Optics within Life Sciences. He is also a Fellow of the Australian Institute of Physics, a Fellow of Optical Society of America, and a Fellow of the International Society for Optical Engineering.

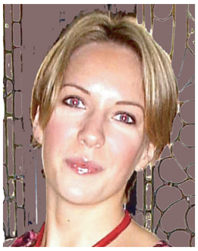

**Sarah Cartmell** (Manchester, UK 1975) was appointed as a new Lecturer in orthopaedic tissue engineering in July 2004. She received a B.Eng. (2 : 1 Hons) degree in materials science with clinical engineering and the Ph.D. degree in clinical engineering (for the thesis: "A degradable bioactive glass: an in vitro and in vivo study") from the University of Liverpool, UK, in 1996 and 2000, respectively. She furthered her studies at Georgia Institute of Technology, Atlanta from 2000–2002 as a Postdoctoral Fellow in orthopaedic tissue engineering. She joined the Institute of Science and Technology in Medicine at the University of Keele in January 2002 where she continued her postdoctoral studies in orthopaedic tissue engineering until obtaining her lectureship position. She has 18 peer-reviewed publications and over 40 published abstracts proceeding from international scientific meetings. She also has given five invited seminars at scientific meetings. She is a Reviewer for several journals and grant applications (EPSRC, BBSRC, UK Department of Health). She is a Member of the Orthopaedic Research Society, the UK Tissue and Cellular Engineering Society, the Tissue Engineering and Regenerative Medicine International Society. Her areas of expertise include bioreactor design, evaluation of biomaterial for orthopaedic applications and mechanotransduction.

